# 30718 Evaluating and advancing the CTSA external advisory board: Best practices

**DOI:** 10.1017/cts.2021.580

**Published:** 2021-03-31

**Authors:** Joseph A. Kotarba, Lori Wiseman, Kevin Wooten

**Affiliations:** University of Texas Medical Branch-Galveston

## Abstract

ABSTRACT IMPACT: The goal of this evaluation study is to enhance the ability of the External Review Board to advise the CTSA at UTMB how to improve translational science activities. OBJECTIVES/GOALS: The purpose of this study is to evaluate the work of the External Review Board (EAB) for the Institute for Translational Sciences/CTSA at the University of Texas Medical Branch-Galveston. This evaluation is conducted through the perceptions of professional and community board members. The outcome consists of an inventory of best practices. METHODS/STUDY POPULATION: We collected data by means of semi-structured interviews with all eight member of the EAB. The interviews were conducted via telephone, lasted approximately 30 minutes each, and were audio-recorded with respondents’ permission. Respondents’ identities were held in confidence. The IRB at UTMB reviewed our study. The interviews were transcribed. The data were analyzed by means of an inductively-oriented, grounded theory approach (Charmaz, 2006). Emergent themes led to the formation of a series of best practices. RESULTS/ANTICIPATED RESULTS: Common concerns included the need for more extensive training for new members; circulation of the agenda before the meeting; and the value of more structured main leadership. The members generally agreed that the breakout groups were valuable because they encouraged them to engage in hands-on responses to practical problems. One of the key epistemological findings was the consensus view that the evaluation of the EAB should be an ongoing project, as opposed to a yearly task. This serious approach to evaluation would be conducive to a process analysis of the EAB, since medical, social, economic, and cultural conditions surrounding and influencing translational science are generally in flux (e.g., the COVID-19 pandemic and the various stages in the CTSA grant). Overall, the EAB experience was quite positive for them. DISCUSSION/SIGNIFICANCE OF FINDINGS: The strongest sentiment expressed in the interviews was that the CTSA at UTMB should focus and build on its strength--the science of team science--as opposed to any concerted search for weaknesses that the term “evaluation” occasionally implies.

